# Hypomagnesemia Causing Convulsions in a Patient Taking a Proton Pump Inhibitor: A Case Report

**DOI:** 10.7759/cureus.76727

**Published:** 2025-01-01

**Authors:** Takanobu Sato, Ryogo Ohashi, Katsuya Konishi, Teuon Cho, Ryo Ichibayashi

**Affiliations:** 1 Division of Emergency Medicine, Department of Internal Medicine, Toho University Sakura Medical Center, Sakura, JPN

**Keywords:** electrolyte abnormalities, esomeprazole, gastric ulcers, hypomagnesemia, proton pump inhibitor

## Abstract

Long-term administration of proton pump inhibitors (PPIs) has been increasingly recognized to cause hypomagnesemia due to impaired magnesium absorption in both the small and large intestines. Hypomagnesemia can lead to various electrolyte imbalances, including hypokalemia, hypocalcemia, and, less commonly, hyponatremia and hypophosphatemia. Measuring serum magnesium levels is essential for identifying the underlying cause in patients receiving PPIs with unexplained electrolyte abnormalities.

Currently, no standardized treatment exists for PPI-induced hypomagnesemia beyond discontinuing PPI therapy and administering magnesium supplements. The main preventive strategies in managing acid-related diseases may be a case-by-case endoscopic evaluation and early discontinuation of PPIs.

This report describes a case of multiple electrolyte abnormalities, including hypomagnesemia, associated with PPI use. It highlights that serum magnesium levels may require several weeks to return to baseline following PPI discontinuation. In outpatient management, repeated serum magnesium measurements over several months are necessary rather than relying on a single assessment post-discontinuation. Furthermore, among the affected electrolytes, magnesium normalization is the most delayed, underscoring the importance of monitoring serum magnesium levels to assess treatment efficacy in cases of complex electrolyte disturbances.

## Introduction

Proton pump inhibitors (PPIs) are drugs widely used to treat diseases involving gastric acid, such as gastric ulcers, duodenal ulcers, and gastroesophageal reflux disease. PPIs have shown superior efficacy in treating acid-related disorders to histamine H_2_ receptor antagonists (H_2_RAs) and have replaced H_2_RAs. The package inserts for PPIs recommend that they be administered for several weeks. Still, in clinical practice, they are often administered for long periods, resulting in side effects associated with long-term use [1].

A typical example is impaired absorption of trace elements. Hypomagnesemia (low magnesium levels) affects the nervous, muscular, cardiovascular, and metabolic systems, causing various symptoms. The mechanism by which long-term PPI use reduces intestinal magnesium absorption and causes hypomagnesemia is gradually being elucidated. This side effect has been reported as an essential PPI-related problem [2-4].

This report describes a case of symptomatic hypomagnesemia, with accompanying hypokalemia (low potassium levels) and hypocalcemia (low calcium levels), caused by the long-term administration of esomeprazole to treat gastric ulcers.

## Case presentation

**Figure 1:**
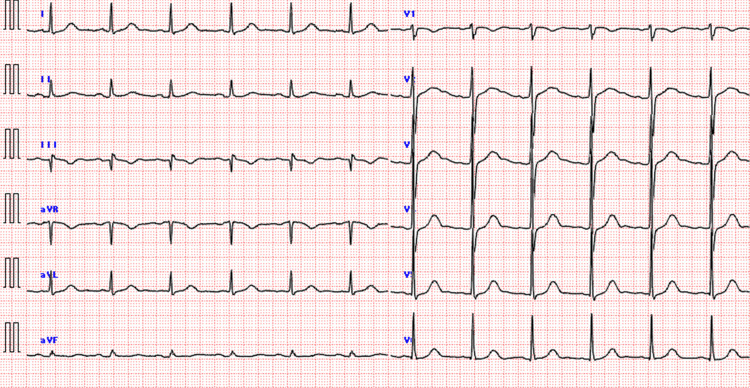
ECG at the time of admission QTc was prolonged to 516 ms.

**Figure 2:**
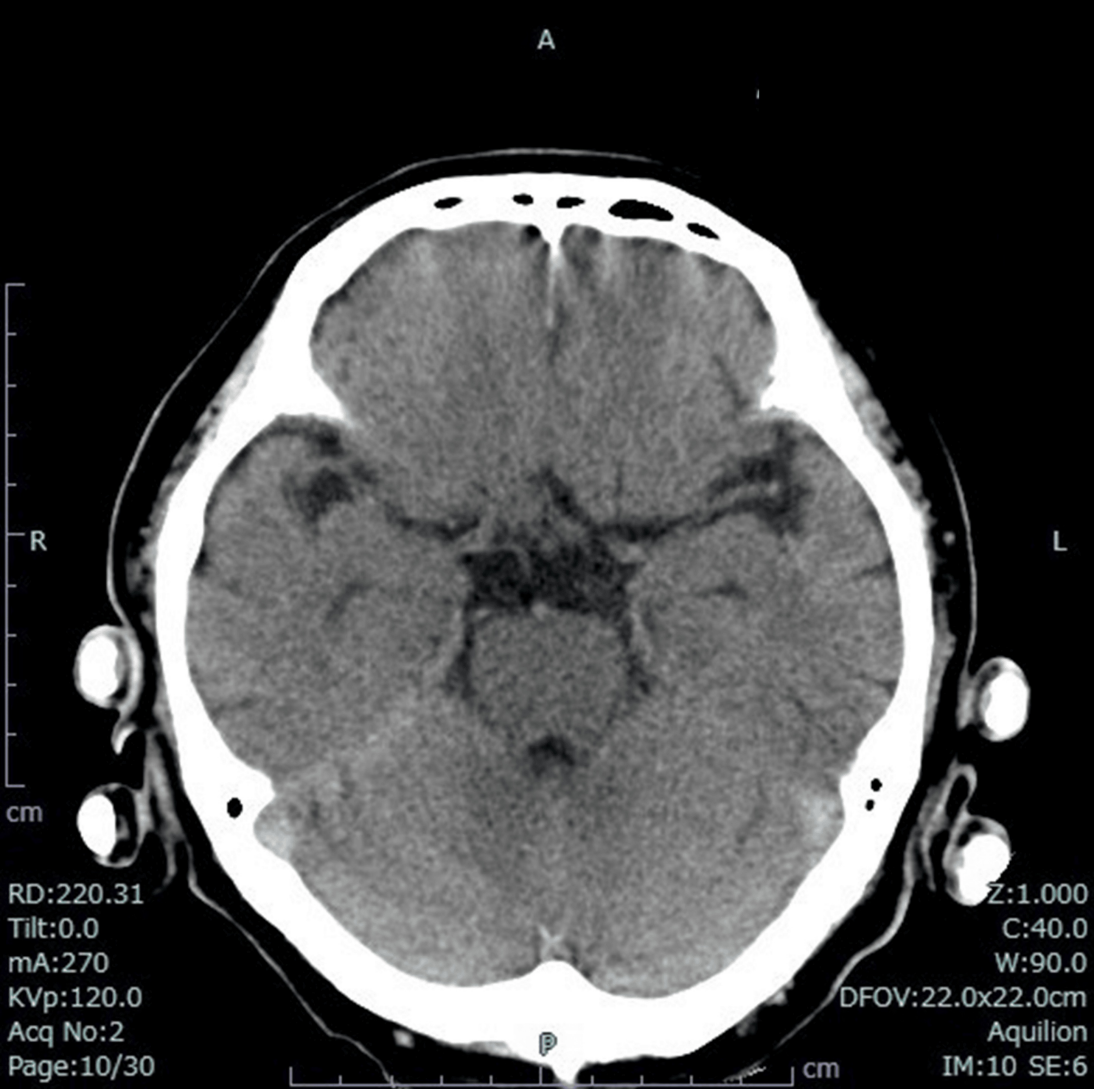
Head CT No abnormal findings.

A 64-year-old man had been treated for prostate cancer, hypertension, and gastric ulcer. He was taking amlodipine, olmesartan, atenolol, tadalafil, naftopidil, esomeprazole, and Miya-BM® (*Clostridium butyricum* MIYAIRI 588 strain; Miyarisan Pharmaceutical Co. Ltd., Tokyo, Japan). He had no history of smoking, and his drinking history was occasional. He had nausea and loss of appetite four days before his visit. As his symptoms continued, he visited a nearby clinic and was prescribed an antiemetic. His symptoms did not improve, and he became dizzy and unable to move, so he called an ambulance. His vital signs at the time of his visit showed that he was drowsy, but he was conscious and had a Glasgow Coma Scale score of 15, blood pressure of 141/100 mmHg, respirations of 29/min, pulse of 111/min, and peripheral oxygen saturation (SpO_2_) of 98%. During the examination, he had generalized tonic convulsions. The convulsions immediately stopped after intravenous injection of 20 mg of diazepam. An ECG showed sinus rhythm, but a prolonged QTc of 516 ms was observed (Figure 1). No abnormalities were found on the head CT (Figure 2). The patient was admitted to the hospital on the same day to be examined for anorexia and seizures. The blood test results at the time of admission are shown in Table 1.

**Table 1:** Laboratory results upon admission CRP: C-reactive protein, TP: total protein, Alb: albumin, AST: aspartate aminotransferase, ALT: alanine aminotransferase, LDH: lactate dehydrogenase, γ-GTP: γ-glutamyl transpeptidase, CK: creatinine kinase, BUN: blood urea nitrogen, eGFR: estimated glomerular filtration, WBC: white blood cell, RBC: red blood cell, Hb: hemoglobin, HbA1c: hemoglobin A1c, Plt: platelet, TSH: thyroid stimulating hormone, FT3: free thyroid 3, FT4: free thyroid 4, PTH: parathyroid hormone, BE: base excess, SG: specific gravity, pCO_2_: partial pressure of carbon dioxide, HCO_3_⁻: bicarbonate, UN: urea nitrogen

Test	Result	Unit	Reference value
CRP	0.26	mg/dL	<0.3
TP	8.5	g/dL	6.7-8.3
Alb	5.0	g/dL	3.8-5.2
AST	41	IU/L	10-40
ALT	27	IU/L	5-45
LDH	323	U/L	124-222
γ-GTP	21	IU/L	<30
CK	1944	IU/L	60-270
BUN	27.5	mg/dL	8.0-20.0
Creatinine	1.7	mg/dL	0.47-0.79
eGFR	33	mL/min/1.73m^2^	>60
Sodium	145	mEq/L	137-147
Potassium	3.1	mEq/L	3.5-5.0
Chlorine	95	mEq/L	98-108
Calcium	8.3	mg/dL	8.4-10.4
Phosphorus	2.6	mg/dL	2.5-4.5
Magnesium	<0.3	mg/dL	1.9-2.5
Glucose	224	mg/dL	70-109
HbA1c	5.7	％	4.7～6.2
WBC	19960	/μL	3300-9000
Hb	14.1	g/dL	13.5-17.5
Plt	36.5	10^4^/μL	14-34
TSH	1.62	μIU/mL	0.35-4.94
FT3	2.91	pg/mL	1.68-3.67
FT4	1.37	ng/dL	0.70-1.48
PTH-intact	89	pg/mL	10-65
Calcitonin	1.30	pg/mL	<5.15
Venous blood gas	-	-	-
pH	7.566	-	7.330-7.410
pCO_2_	24.2	mmHg	43-53
HCO_3_⁻	21.5	mmol/L	24-28
BE	1.2	mmol/L	0-4
Lactate	-	-	-
Urinalysis	-	-	-
pH	7.5	-	5.0～8.0
SG	1.025	-	1.010～1.025
UN	308	-	-
Creatinine	315.98	-	-
Sodium	30	mEq/L	-
Potassium	71.7	mEq/L	-
Magnesium	0.5	mg/dL	-
WBC	5-9	/HPF	-
Bacteria	(1+)	-	(-)

Blood tests showed hypokalemia, hypocalcemia, and hypomagnesemia. Other findings included elevated creatinine kinase (CK) and white blood cell (WBC) count. Although there was no elevation in C-reactive protein (CRP), bacteriuria and abdominal CT showed elevated perirenal fat tissue density on both sides, leading to a diagnosis of urinary tract infection. No abnormal findings were found on the MRI or EEG. Therefore, the cause of the convulsions, nausea, dizziness, and loss of appetite was diagnosed as severe hypomagnesemia. On the first day of hospitalization, 20 mEq of magnesium sulfate was administered intravenously in one shot, followed by continuous administration of 40 mEq of magnesium sulfate over 24 hours to correct the condition slowly. On the second day of hospitalization, serum magnesium had improved to 2.1 mg/dL, and symptoms had improved. Since the uncorrected fractional excretion of magnesium (FEMg) was 1%, it was determined to be an extrarenal loss. The cause of hypomagnesemia was suspected to be oral esomeprazole. On the fourth day of hospitalization, upper gastrointestinal endoscopy was performed, and no abnormal findings were found in the esophagus, stomach, or duodenum. Therefore, oral administration of esomeprazole was discontinued. Hypokalemia and hypocalcemia improved naturally without the need for special correction. On the fifth day of hospitalization, serum magnesium temporarily decreased to 1.7 mg/dL, and 20 mEq of magnesium sulfate was administered. After confirming the increase in CK associated with convulsions and the disappearance of symptoms, the patient was discharged on the seventh day of hospitalization. Seven days after discharge, the patient developed diarrhea, and serum magnesium decreased to 1.5 mg/dL, but no oral treatment was administered. Eighteen days after discharge, diarrhea improved, and serum magnesium was 1.9 mg/dL. No correction was performed thereafter, and serum magnesium was 1.8 mg/dL 28 days after discharge, maintaining typical values. The progress of blood electrolyte test results is shown in Table 2.

**Table 2:** Electrolyte course of blood tests during hospitalization and after discharge This table presents laboratory data and esomeprazole administration details from the day of hospitalization (Day 1) to the post-discharge follow-up (Day 35). Serum magnesium levels: Hypomagnesemia was prominent on Day 1, and gradual recovery was observed after discontinuing esomeprazole. Serum potassium concentration: Hypokalemia was observed initially, improving with treatment. FEMg: Fractional excretion of magnesium

	Hospitalization					After discharge			Reference value
	Day 1	Day 2	Day 3	Day 5	Day 7	Day 14	Day 25	Day 35	-
Esomeprazole	(+)	(+)	(+)	(−)	(−)	(−)	(−)	(−)	-
Magnesium sulfate administration (g)	7.38	0	0	2.46	1.32	1.32	0	0	-
Magnesium (mg/dL)	<0.3	2.1	1.9	1.7	1.5	1.5	1.9	1.8	1.9-2.5
Corrected calcium (mg/dL)	8.3	8.1	8.1	9.1	9.6	9.9	9.5	9.3	8.4-10.4
Sodium (mEq/L)	145	142	142	139	141	141	140	143	137-147
Potassium (mEq/L)	3.1	2.8	3	3.8	4.2	3.7	4.2	3.7	3.5-5.0
Phosphorus (mg/dL)	2.6	3	2.3	1.7	1.4	2.9	2.9	3.1	2.5-4.5
Creatinine	1.7	1.09	0.76	0.68	0.68	0.83	0.87	0.89	0.47-0.79
FEMg (%)	1	-	-	-	-	-	-	1	-

**Figure 3:**
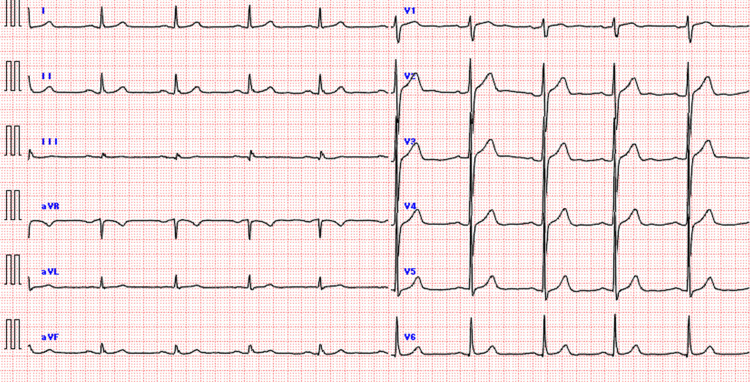
ECG after treatment The QTc interval improved to 419 ms.

The ECG showed an improvement in the QTc interval to 419 ms, and the patient ceased outpatient treatment (Figure 3). Based on the above history and findings, it was diagnosed that the oral administration of esomeprazole caused hypomagnesemia.

## Discussion

Approximately 30% to 50% of daily magnesium intake is absorbed in the digestive tract. Notably, there is a negative correlation between intake and absorption efficiency - the higher the intake, the lower the absorption rate [3]. Magnesium absorption in the intestinal tract occurs through two distinct pathways: the paracellular and transcellular pathways. In the distal jejunum and ileum of the small intestine, magnesium is primarily absorbed via the paracellular pathway. This process involves the diffusion of magnesium ions between epithelial cells through tight junctions, a mechanism regulated by cation-selective proteins such as claudin 2 (CLDN2), CLDN7, and CLDN12. In contrast, the transcellular pathway facilitates magnesium absorption in the large intestine. This pathway relies on specific channels and transport proteins. On the luminal side, transient receptor potential M6 (TRPM6) and TRPM7 channels enable magnesium ions to enter the epithelial cells. Subsequently, cyclin M4 (CNNM4) on the basolateral side ensures the transport of magnesium into the bloodstream, completing the absorption process. This integrated mechanism highlights these pathways' distinct yet complementary roles in maintaining magnesium homeostasis within the body [3,4].

PPIs may increase intestinal pH, reducing the solubility of magnesium and thereby decreasing its absorption efficiency. Additionally, PPIs are thought to increase transepithelial electrical resistance (TEER) and reduce paracellular permeability, further impairing magnesium absorption in the small intestine [3,4]. Elevated intestinal pH due to PPIs may also affect TRPM6/7 channel activity in the large intestine, disrupting transcellular magnesium transport [3,4]. Moreover, PPI-induced reductions in gut microbiota diversity may hinder bacterial fermentation in the colon, preventing acidification and further compromising magnesium absorption [3].

The time to onset of PPI-induced hypomagnesemia varies significantly among individuals, with a median onset of 5.5 years. However, reported onset times range widely from 14 days to 13 years, making it challenging to predict hypomagnesemia based on the duration of PPI use alone [5]. In this case, the 12-year duration of PPI administration exceeds the median, highlighting the unpredictable nature of this adverse effect.

Currently, there is no established treatment for PPI-induced hypomagnesemia beyond discontinuing PPIs and administering magnesium supplementation [3]. Serum magnesium levels guide dosage adjustments in patients with stable hemodynamics. If renal function is normal and serum magnesium levels are <1.0 mg/dL (0.8 mEq/L), 1-2 g of magnesium sulfate (8-16 mEq of magnesium) is administered over 30-60 minutes, followed by 4-8 g of magnesium sulfate (32-64 mEq of magnesium) infused slowly over 12-24 hours [6]. Administration continues until serum magnesium levels reach ≥1.0 mg/dL. If serum magnesium levels are ≥1.0 mg/dL (0.8 mEq/L), 4-8 g of magnesium sulfate (32-64 mEq of magnesium) is administered slowly over 12-24 hours [6].

In this case, serum magnesium levels were <0.3 mg/dL initially. As an outpatient, the patient received 2.46 g of magnesium sulfate over 60 minutes, which increased serum magnesium levels to 1.2 mg/dL. Upon hospitalization, 4.92 g of magnesium sulfate was administered continuously over 24 hours on the first day. On days 2, 5, and 7, and after discharge on day 14, 1.23-2.46 g of magnesium sulfate was administered repeatedly over 60 minutes to achieve a target serum magnesium level of ≥1.9 mg/dL. As a result, serum magnesium levels normalized on the 25th day of hospitalization. The half-life of repeated oral administration of 10 mg of esomeprazole is approximately 1.16 hours, and its blood concentration is assumed to approach zero within 24 hours of discontinuation. However, despite discontinuing esomeprazole on the fourth day of hospitalization, it took approximately three weeks for serum magnesium levels to normalize. Since the patient could consume adequate food during hospitalization and had no other medications or factors contributing to hypomagnesemia, intestinal magnesium absorption may take several weeks to recover even after esomeprazole is eliminated from the body.

In a study comparing the time to relapse of hypomagnesemia after resuming PPIs - including omeprazole, esomeprazole, pantoprazole, lansoprazole, and rabeprazole - no significant difference was observed among these drugs. This finding suggests that switching between PPIs is not effective in preventing hypomagnesemia [5]. Conversely, it remains controversial whether switching to a different antacid, such as an H_2_RAs, offers any benefit [5]. It has been reported that prebiotics and probiotics might help prevent PPI-induced mineral deficiencies [7]. However, in this case, a butyric acid preparation was already being used alongside PPIs before hospitalization, suggesting limited effectiveness in preventing electrolyte abnormalities. Given these observations, early discontinuation of PPIs combined with a case-by-case gastrointestinal endoscopy is currently considered the only reliable preventive strategy against PPI-induced hypomagnesemia. Hypomagnesemia can lead to hypokalemia, hypocalcemia, and, in rare cases, hypophosphatemia and hyponatremia [2]. Therefore, measuring serum magnesium levels in long-term PPI therapy patients with hypokalemia, hypocalcemia, or other unexplained electrolyte imbalances is crucial.

Potassium is transported into cells via the sodium-potassium adenosine triphosphatase (Na⁺-K⁺-ATPase) pump in the distal tubule and collecting duct of the kidney. It is then secreted into the tubular lumen through two potassium channels: renal outer medullary potassium (ROMK) and big potassium (BK) channels [8]. Magnesium normally inhibits the ROMK channel. In cases of hypomagnesemia, this inhibition is lost, leading to persistent potassium secretion through the ROMK channel and subsequent hypokalemia. As a result, hypokalemia associated with hypomagnesemia is often resistant to potassium supplementation alone, and magnesium replacement is typically required to correct the imbalance [8].

Additionally, hypomagnesemia can cause functional hypoparathyroidism [9]. When serum magnesium levels decrease, the α subunit of the G protein (Gα protein) is inhibited, leading to activation of the calcium-sensing receptor (CaSR) in the parathyroid gland and subsequent suppression of parathyroid hormone (PTH) secretion [10]. Intravenous administration of magnesium has been shown to increase PTH secretion rapidly within minutes [10]. In this case, PTH levels were measured on the second day of hospitalization, after magnesium had already been administered, which may explain the slightly elevated PTH level compared to baseline. Furthermore, magnesium acts as a cofactor for adenylate cyclase, and its deficiency can disrupt signal transduction at the parathyroid hormone 1 receptor (PTH1R), contributing to hypocalcemia [11].

In this patient, serum potassium and calcium levels improved shortly after discontinuing PPI therapy and normalized by the fifth day of hospitalization. However, serum magnesium levels required longer to normalize, underscoring the prolonged recovery period for magnesium homeostasis after chronic PPI exposure. Indeed, it is essential to continue monitoring serum magnesium levels for several months following PPI discontinuation as long-term electrolyte replacement may be necessary to replenish body stores fully [2].

## Conclusions

This case highlights convulsive symptoms resulting from severe hypomagnesemia induced by long-term oral administration of esomeprazole. The underlying mechanism of PPI-induced hypomagnesemia involves impaired magnesium absorption, alterations in intestinal flora, and subsequent electrolyte imbalances. These findings emphasize the importance of regular serum magnesium monitoring in patients undergoing long-term PPI therapy. Additionally, unnecessarily prolonged use of PPIs should be avoided to mitigate the risk of potentially severe electrolyte abnormalities and associated complications.
